# Know and grow: a qualitative evaluation of a parent skills training intervention

**DOI:** 10.1186/2050-2974-2-S1-O36

**Published:** 2014-11-24

**Authors:** Gabrielle Goodier, Julie McCormack, Sarah Egan, Hunna Watson, Gillian Todd, Janet Treasure, Kimberley Hoiles, Sue Lister, Kaye James

**Affiliations:** 1 School of Psychology and Speech Pathology, Curtin University, Perth, Australia; 2 Specialised Child and Adolescent Mental Health Service, Princess Margaret Hospital for Children, Perth, Australia; 3 South London and Maudsley NHS Trust, London, UK; 4 Psychological Medicine Department, King's College London, London, UK; 5 Institute of Psychiatry, London, UK

## Objective

This qualitative study examined the experience of parents of children and adolescents with eating disorders after having participated in a skills-based training intervention.

## Method

Participants were interviewed and transcripts were analysed using inductive thematic analysis.

## Results

Parent responses were organised around key themes of (1) effectiveness and acceptability of the intervention; (2) interpersonal experience of the group process; and (3) feedback on intervention content. Overall, the program was seen by parents to be highly relevant with direct application to supporting their child in home and hospital environments.

## Discussion

This study reports on preliminary evidence that skills-based training is acceptable to parents and improves parent functioning including parent self-efficacy, and reduces psychological distress, anxiety, and burden. The study also demonstrated that the intervention can be delivered in a tertiary paediatric treatment setting and it may become cost-effective method for supporting parents and other carers. Future research is required on treatment efficacy and patient outcomes.

This abstract was presented in the **Parental Roles in Prevention and Support** stream of the 2014 ANZAED Conference.

